# Arab American women and the generational cycle of shame: A cognitive reading of Etaf Rum’s *A Woman Is No Man*

**DOI:** 10.1111/oli.12321

**Published:** 2021-09-03

**Authors:** Marijana Mikić

**Affiliations:** ^1^ University of Klagenfurt Klagenfurt Austria

**Keywords:** Arab American literature, cognitive narratology, emotion, gender, shame

## Abstract

The essay uses a cognitive narratological approach to analyze how Etaf Rum’s *A Woman Is No Man* (2019) negotiates Arab American patriarchal culture through the lens of shame. By narrating the emotional experiences of Arab American women who bear the pain of shame while they also engage in shaming others, Rum gives readers the opportunity to understand better the complex relationship between the psychology of shame and the “shame of gender” in patriarchal Arab American culture. Not only does *A Woman Is No Man* articulate the lived experiences of one of the most “forgotten” and silenced ethnic groups within American literature and culture, it also draws attention to how gender‐based shaming is shaped by, and contributes to shaping, a culture of patriarchy and male power. The novel uncovers the different ways in which shame impacts the minds and bodies of Arab American women across three generations, while also laying bare the psychological, gendered, and socio‐culturally embedded aspects that shape the elicitation, experience, expression, and regulation of shame.

## 1 INTRODUCTION

There is an unspoken rule, common among ethnic American writers, never to “air their dirty laundry” in public, which has become arguably even more important for Middle Eastern and Muslim American writers following the events of 9/11. The opening of Etaf Rum’s *A Woman Is No Man* (2019) defines this contract of silence as a specifically female condition:

Where I come from, voicelessness is the condition of my gender, as normal as the bosoms on a woman’s chest, as necessary as the next generation growing inside her belly. But we will never tell you this, of course. Where I come from, we’ve learned to conceal our condition. We’ve been taught to silence ourselves, that our silence will save us. It is only now, many years later, that I know this to be false. Only now, as I write this story, do I feel my voice coming. (Rum, 2019b, 1)

Not only does Rum point to the danger of defying community rules, she uses these words to mark the beginning of her own storytelling and to communicate that breaking long‐held silences is important and necessary. Her semi‐autobiographical debut novel takes an unflinching look at how patriarchal gender role expectations and gender‐based family violence impact three generations of Arab American women: Isra, born and raised in Palestine and married off at the age of seventeen to a Palestinian American man, who tragically beats her to death after she tries to escape familial abuse and shaming; Isra’s daughter Deya, who, born and raised in the U.S., defies gender‐based expectations of marriage and motherhood; and Fareeda, who becomes complicit in familial gender oppression by enforcing the patriarchal norms of traditional Arab culture. By inviting her readers to share the thoughts and emotions of her three different character focalizers along two alternating timelines—1990 and 2008—Rum transcends time and place to ensure that their stories no longer remain unheard.

In an interview, Rum reveals that one of the biggest challenges in writing *A Woman Is No Man* was the fear of confirming “the many stereotypes that exist about Arab culture, specifically that every man is violent and every woman is a victim” (Rum, 2019a). Her literary representation of abuse and oppression indeed runs the risk of playing into the already pervasive stigmatization of Arab and Muslim Americans—particularly through its dangerously generalizing and often one‐dimensional portrayal of abusive male characters. However, it is her focus on the *subjectivity* of Arab American women, and the fact that readers see these male characters through the eyes of their mothers, wives, and daughters, that offers what Chielozona Eze has called “the healing power and moral obligations of testimonies” (Eze, 2014, 288). Testimonializing “the shame and nostalgia inherent in being *bint/ibn Arab* (daughter/son of Arabs),” while simultaneously transcending “such limited and limiting conceptualizations of identity and heritage” (Fadda‐Conrey, 2014, 19) is the moral and ethical imperative of *A Woman Is No Man*.

This essay uses a cognitive narratological approach to analyze how Rum’s *A Woman Is No Man* negotiates Arab American patriarchal culture through the lens of shame. By narrating the emotional experiences of Arab American women who bear the pain of shame while they also engage in shaming others, Rum gives readers the opportunity to understand better the complex relationship between the psychology of shame and the “shame of gender” in patriarchal Arab American culture. My reading of the novel ties in with recent research by scholars such as Frederick Luis Aldama, Stephanie Fetta, Sue J. Kim, and Alexa Weik von Mossner, whose work has been instrumental for creating a dialogue between cognitive narratological approaches to emotion in literature and the study of ethnic American fictions.^1^ Not only does *A Woman Is No Man* articulate the lived experiences of one of the most “forgotten” and silenced ethnic groups within American literature and culture, it also draws attention to how gender‐based shaming is shaped by, and contributes to shaping, a culture of patriarchy and male power. The novel uncovers the different ways in which shame impacts the minds and bodies of Arab American women across three generations while also laying bare the psychological, gendered, and socio‐culturally embedded aspects that shape the elicitation of shame as well as emotion regulation and behavioral response strategies.

## 2 THE PSYCHOLOGY OF SHAME AND CONSERVATIVE ARAB AMERICAN GENDER ROLE EXPECTATIONS

Shame is a complex, negative emotion which is evoked by self‐evaluation. Together with other self‐conscious and negative emotions, such as guilt, shame “function[s] as an emotional moral barometer, providing immediate and salient feedback on our social and moral acceptability” (Tangney et al., 2007, 347). But in contrast to emotional experiences of guilt, which involve “a negative evaluation of a specific behavior,” shame “involves a negative evaluation of the global self” (p. 349). Shame, according to psychologists Kemeny, Gruenewald, and Dickerson, is thus “a key emotional response to events in which the positive value or status of one’s *social self* is threatened” (Kemeny, Gruenewald, & Dickerson, 2004, p. 154, emphasis added). According to developmental psychologist Michael Lewis, typical phenomenological responses to shame include the desire to “hide, disappear, or die” (Lewis, 2008, 748). The exposure of the self thus creates a sense of worthlessness which is most typically embodied through the lowering of one’s head, shrinking of one’s body, or the inability to speak. While shame, as a threat to the self, is individually experienced, its elicitation is tied to processes of socialization that impact the “social self.” As such, shame has routinely been used to enforce “the ethics of community” (Tangney et al., 2007, 348).

Indeed, the usage of shame as a tool of socialization has always had a special place in conservative Arab (American) culture and its enforcement of a patriarchal social system. Rita Stephan and Mireille Aprahamian argue that patriarchal structures are important elements of Arab American communities in which

women are often expected to maintain family unity and privacy. Stepping outside these traditional roles places Arab American women in a difficult position among their family centered communities. They are viewed as if they have co‐opted the “American ways” and lost their cultural values and family ties. (Naber, 2012, 120)

Arab American women who defy traditional cultural expectations and/or get “Americanized”—by engaging in pre‐/extra‐marital “sexual behavior, asking for a divorce, challenging men’s authority, or criticizing one’s husband” (Vang, Cuevas, Sharma, & Rueda, 2018, 158)—are often seen as shaming the family. The strong focus on the family, which extends the impact of “individual shameful behaviors [. . . to] the entire family unit” (p. 158), also points to a stark contrast between American individualism and the high collectivism of conservative Arab cultures which rely on practices of shaming and dishonoring to enforce societal and gender‐related rules.

The social and gendered implications of the Arab concept of honor might even be exacerbated after geographical relocation, as Nadine Naber points out:

The concept of *al‐nas* [the people] takes on new form in the diaspora. It works as shorthand for an imagined Arab community. It enhances the possibilities for the politics of cultural authenticity to regulate behaviors by rendering transgressions not only as individual rebellion but as cultural loss and Americanization. The power of *al‐nas* in America is that it means that one’s behavior implicate one’s entire family as well an entire “Arab people” in the United States. (Naber, 2012, 101)

While the preservation of traditional Arab culture is typical for “the first generation which tends to follow tradition more uncritically as a way to cling to their countries of origin,” Marta Bosch‐Vilarrubias suggests that “second‐generation individuals accept this [traditional] inheritance and choose whether to follow it or not” (Bosch‐Vilarrubias, 2016, 74). An understanding of the diasporic experiences of Arabs in the U.S. thus demands a recognition of the underlying hybridity of identity created by tensions between being both Arab *and* American. Whereas first generations tend to prefer national‐origin identities, subsequent generations are more likely to adopt hybrid identities, which may create intergenerational conflicts, as *A Woman Is No Man* illustrates.

Rum’s literary representation of cross‐generational issues is not centrally focused on the intersecting forms of oppression that Arab American women face “as the racial, religious, political, and national Other” (Fadda‐Conrey, 2014, 1) within hegemonic U.S. American culture. Instead, the novel negotiates how gendered practices of inter‐community shaming are shaped by, and contribute to shape, patriarchal systems of oppression. Through her use of variable focalization, Rum not only represents the generational cycle of shame—and the psychological and physical suffering of different Arab American women—her narrative also highlights the diverging strategies of emotion regulation and response which contribute to define the characters’ cross‐generational progression from withdrawal, internalization, and complicity to resistance and healing.

## 3 READING SHAME AND GENDER IN *A WOMAN IS NO MAN*

The novel first takes readers to Birzeit, Palestine, and seventeen‐year‐old Isra Hadid who dreams “of not being forced to conform to conventions, of adventure, and most of all, of love” (Rum, 2019b, 9). Her mother, however, quickly blasts her hopeful notions of love and life—that she picked up as an avid reader of romantic novels—reminding her that “There is nothing out there for a woman but her *bayt wa dar*, her house and home. Marriage, motherhood—*that* is a woman’s only worth” (p. 11). Rum immediately establishes the cultural environment as hyper‐patriarchal with strong predetermined gender roles that expect every woman to follow the path of “respectability” by being a wife and a mother. The dutiful fulfillment of these roles is, in turn, connected to her “worth,” that is to say, her social status within the community. When Isra’s parents decide to marry her off to a Palestinian American man, Adam Ra’ad, she is afraid of what the future has in store for her, but moving to the United States makes her hopeful that in “the land of the free [. . .] perhaps she could have the love she had always dreamed of, could lead a better life than her mother’s” (p. 21). While Rum’s early portrayal of her character negotiates common and stereotypical understandings of romance, love, and American freedom, she does so for purposes of critique: The stories that Isra has read all her life do not portray characters whose life experiences resemble hers. Rum’s own novel, of course, seeks to address this lacuna by giving a voice to silenced Arab American women.

Isra’s new life in Brooklyn, New York, with her husband Adam and his family soon turns into a tragic story devoid of self‐determination and happiness. She is far away from home, but her mother’s words that a woman’s place is in the home still ring true, and quite literally so, since Isra is barely allowed to leave her family’s house in Brooklyn’s Bay Ridge neighborhood. Isra tries to win her husband’s love, but she recoils from Adam’s touch, who is still a stranger to her. And when she gives birth to their first child—a girl—her new family does little to hide their discontent about the baby’s gender and their disappointment in the baby’s mother. In a culture that traditionally defines a daughter as only “a temporary guest, quietly awaiting another man to scoop her away, along with all her financial burden” (Rum, 2019b, 6), the disappointment of having one is, unsurprisingly, only directed at the mother. The first few moments after birth make Isra’s heart swell with happiness, but soon after her mother‐in‐law, Fareeda, makes Isra experience the painful feeling of shame at her inability to give Adam a son, whispering: “If you don’t give a man a son, he’ll find him a woman who can” (p. 110). Through Fareeda’s interaction with Isra, the narrative negotiates “pride and shame [. . . as] background emotions for self‐appraisals in the ‘looking glass’ provided by others’ responses to a person’s behaviors” (Stets & Turner, 2008, 32). The perceived threat to family pride, which is highly valued within the Arab American community, moves Fareeda to shame Isra for her apparent ineptitude to properly fulfill her duty as a wife, daughter‐in‐law, and, more broadly, worthy member of the family. Because the looking glass is typically directed at the woman, Adam remains exempt from familial shaming.

Even though Rum only gives readers insight into Isra’s consciousness—and never into Adam’s—she negotiates the roles of traditional gendered expectations through interactions between them. On the one hand, Adam enjoys male power and privilege which put him in a position to give Isra orders on how to behave within and outside their home, cautioning her that the family’s reputation within the (insular) Arab American community depends on her demeanor. On the other hand, it becomes clear that he, too, is pressured to conform to the traditional gendered expectations for Arab American men, which include financially supporting the family and producing male heirs who can carry on the family line. His rare conversations with Isra reveal his struggle: “What’s there to be happy about? [. . .] ‘Do this, Adam! Do that, Adam! More money! We need a grandson!’ I’m doing everything I can to please my parents, but no matter what I do, I fall short” (Rum, 2019b, 146). Through his frequently blood‐shot eyes it also becomes clear that he uses alcohol as a maladaptive coping strategy.^2^ Although Adam is not routinely shamed by his family, his fear of failing to adhere to traditional gendered expectations of patriarchal masculinity likely makes him feel shame all the same and it subsequently also exacerbates his anger toward Isra.

As the novel progresses, and Isra gives birth to three more children—all of them girls—the pressure to produce a male heir becomes a suffocating presence in the family’s life: “Not a day had passed when Fareeda had not mentioned the child’s gender, how they needed a grandson, how Isra had disgraced them in the community” (Rum, 2019b, 157). Isra, who has become accustomed to the constant shaming by Adam’s mother and the silence between herself and her husband “learned to shrink herself in his presence so as not to upset him” (p. 171). As mentioned earlier, the phenomenological experience of shame typically creates a desire in the shamed person to hide, which is why “the physical action accompanying shame is a shrinking of the body, as though to disappear from the eye of the self or the other” (Lewis, 2008, 748). Indeed, Fareeda’s constant shaming causes Isra to experience the pain of worthlessness and to withdraw from both life and religion. While she has been trying to find a way to cope with her debilitating emotions through faith, she increasingly feels that “she had nothing to say to God” (Rum, 2019b, 169). In *The Oxford Handbook of Religion and Emotion* (2008), Anna M. Gade points out that the “Qur’an instructs that created beings are in an emotional relationship to their Creator, obliged to him to feel emotions such as thankfulness and adoration [. . .] and patiently anticipating God’s judgment” (Gade, 2008, 37). The culturally enforced and gendered “obligation” to bear the blame for her family’s unhappiness, however, causes Isra to feel crippling feelings of powerlessness that prevent her from believing in her worth before God.

When the wife of Adam’s younger brother gives birth to a son, Isra knows that she can no longer hide in plain sight of her husband. Whereas Isra’s reaction to shame is withdrawal, Adam reacts with anger and physical violence against his wife. Tangney et al. point out that “research indicates a robust link between shame and anger,” which suggests that “shame‐prone individuals are more likely to engage in externalization of blame, experience intense anger, and express that anger in destructive ways, including direct physical, verbal, and symbolic aggression” (Tangney et al., 2007, 351). That night, Adam physically harms his wife for the first time and soon his physical abuse becomes routine for Isra, who has already internalized her shame to such a degree that she accepts her husband’s violence as “her way for apologizing for all she had done” (Rum, 2019b, 183). Even though everyone in the household is aware of the physical abuse, nobody does anything to help Isra. On the one hand, the representation of Adam’s aggression and violence plays into stereotypical definitions about Arab American masculinity which have become even more prevalent with the rise of post‐9/11, anti‐Middle East, and anti‐Muslim rhetoric. On the other hand, the normalization of Adam’s destructive anger and physical aggression in his own family environment lays bare the ways in which strategies of emotion regulation and response are shaped by and contribute to maintain patriarchal social orders. Brody, Hall, and Stokes suggest:

The socialization of emotion is especially influenced by characteristics of the family system, including the parents’ own temperaments, their gender role attitudes and behaviors, the quality of their marital relationships, their cultural and socioeconomic backgrounds, and the gender constellation of the children in their families. (Brody et al., 2016, 384)

Adam’s response of aggression—likely based on his own witnessing of other men’s violence and habitual shaming of women—is therefore not merely an individual abusive act, but it is reflective of a socio‐cultural environment that normalizes gender‐based oppression and abuse of women.

The socialization of emotions according to traditionally patriarchal notions, in turn, results in harmful practices of female victim blaming. Beitin and Aprahamian argue that “values of honor and shame are related to women’s tolerance of violence against them,” which means that “women often tolerate abuse to protect family privacy and cohesion” (Beitin & Aprahamian, 2013, 71). This is shown in the novel, for example, when Fareeda scolds Isra for coming to breakfast with a bruise on her cheek, telling her how she “never let anyone see my shame” (Rum, 2019b, 183). Even though Fareeda was abused herself, she allows her son to continue abusing his wife. But covering up the bruises and “pretending only worked on the outside. Inside, Isra was filled with a paralyzing shame. She knew there must be something dark stemming from within her to make the men in her life do these terrible things” (p. 185). Through the representation of Isra’s experience, Rum negotiates what Lewis calls “the global attack on the self‐system” that is caused by the emotional state of shame (Lewis, 2008, 748). The claustrophobic circumstances of Isra’s life evoke emotions of “anger, resentment, shame, despair” that make her want “to scream” (Rum, 2018b, 147). But her embodied conviction of female worthlessness, and the accompanying objectification and otherization of her own self, shame her into silence.

As time goes by, Isra’s individual shame morphs into collective, cross‐generational shame as the insidious effects of her own psychological and physical abuse begin to impact her relationship on her daughters. After the birth of her second child, she cannot but acknowledge “that she was not particularly motherly” (Rum, 2019b, 147); and in her daughters’ eyes she sees that “they were judging her” for being unable to show her love (p. 186). The devastating mental and bodily experience of being inherently flawed—caused by her constant exposure to gender‐based shaming—and the culture of female passivity that she has been taught all her life are so deeply engrained in Isra that

even though she willed herself not to, she secretly resented her daughters for being girls, couldn’t even look at them without stirring up shame. She wanted to say that it was a shame that had been passed down to her and cultivated in her since she was in the womb, that she couldn’t shake it off even if she tried. (p. 301)

Here, Isra’s thoughts highlight how shame may not only be internalized but how the effects of shame‐based trauma may also be passed down intergenerationally.^3^ While she has been shamed all her life, Isra is also becoming implicated in the cycle of oppression that would make her daughters live a “life [that] was nothing but a dark melody [. . .] and she was to blame” (p. 248). The consequences of Isra’s experiences of shame not only lead to her emotional withdrawal from her daughters, but they also stir up intense feelings of guilt. The eyes of her first daughter, Deya, “haunted Isra the most. [. . .] The sight was intolerable, but Isra didn’t know how to make it go away” (p. 186). Shame and guilt are both self‐conscious and negative emotions, but Tangney et al. remind us that in contrast to shame, which is focused on ”concern with others' evaluations of the self,” someone experiencing guilt is ”more likely to recognize (and have concerns about) the effects of that behavior on others rather than on others’ evaluations” (Tangney et al., 2007, 349). Isra’s painful feelings of guilt arise from her inability to speak up and save her daughters from the path that traditional Arab culture prescribes for women even though she desperately wishes to teach her daughters “how to love themselves, [knowing] that this was the only way they had a chance at happiness” (Rum, 2019b, 250). While Isra’s experiences of shame have evoked reactions of withdrawal, her subsequent behavioral response to her own moral failings as a parent corresponds to well‐known action tendencies associated with guilt.

Isra’s attempt to run away with her daughters is driven by her guilt which, according to Tangney et al., promotes “constructive, proactive pursuits,” invested in “undoing the consequences of the behavior” (Tangney et al., 2007, 350). Her decision to escape signifies a reparative action that is directed at both herself and her daughters. The newfound determination enables her to (re)locate a self that exists outside her internalized sense of female worthlessness and to act with the resolution to save her daughters from a culture whose shared values threaten and undermine the female sense of self. Having made her decision, Isra is aware that “There was no turning back now. If Adam knew she was running away, if he found her now, he would beat her to death. She was sure of it. But it didn’t matter. She had made her choice” (Rum, 2019b, 337). Isra’s storyline and the novel itself end on this hopeful note, but readers in fact have learned much earlier in the novel—through Rum’s use of alternating timelines and variable character focalizers—that Isra and her daughters later encounter Adam in the subway. Readers know that they leave the subway together and have a last picnic in the park as a family before Adam murders Isra for trying to run away and subsequently commits suicide. Even though the event of Isra’s death points to the tragic consequences of her self‐determined behavior, Rum’s choice to end Isra’s storyline—and the novel—with the words “They would finally be free” (p. 337) as Isra and her daughters are waiting for the subway train to arrive seems crucial to the desired political efficacy of her novel. Rum (re)articulates and (re)imagines the subjectivity of Arab American women through each individual storyline, but she also interweaves the storylines of her characters to call for solidarity amongst Arab American women to share their stories and form “communities of resistance” (Mohanty, 2003, p. 47).

In the second major storyline—set eighteen years later in Brooklyn—Isra’s daughter Deya describes the day of Isra’s death as the last memory of her parents. Deya remembers how the whole family went to the park together after she, her mother, and her sisters ran into their father in the subway. Although Deya knew nothing of her mother’s escape plans, and her father’s discovery thereof, readers learn “that day in the park, staring at her parents at opposite ends of the blanket, she’d felt as though she understood the meaning of the word *sorrow* for the first time” (Rum, 2019b, 43). No words are exchanged, but her parents’ embodiment of sorrow evokes a foreboding feeling in Deya which foreshadows Isra’s murder and Adam’s subsequent suicide. Deya cannot reconcile this memory with her memory of the events preceding the situation in the park when her mother was “smiling wider than [. . . Deya] had ever seen” (p. 219). By focalizing the event of the attempted escape through Deya’s consciousness, the narrative allows readers to see that Isra was speaking resistance with her whole body although she never verbalized her plans to anyone. All her life, Fareeda has made Deya believe that her parents died in a car accident, but when Deya comes into possession of a newspaper clipping, which reports that her mother was murdered by her father, this presents a pivotal moment in her life and in the novel.

When readers are introduced to Deya, she is in the process of meeting potential suitors to have her marriage arranged in much the same way her mother did all those years ago. Raised by her grandmother Fareeda, Deya is taught that “Preserving our culture is what’s most important” (Rum, 2019b, 26). Since the preservation of patriarchal culture goes hand in hand with marriage and motherhood, she is set to get married as soon as she finishes high school. Deya, however, manages, to scare off all her suitors, who think that she is “too insolent, too questioning. That she wasn’t Arab enough” (p. 28). Typical of second‐ and third‐generation immigrants, Deya occupies a space of hybridity. Amal Talaat Abdelrazek suggests that in “this space hybridized individuals erase any claims for inherent cultural purity and inhabit the realm of an in‐between reality marked by shifting psychic and cultural boundaries (Abdelrazek, 2007, 69). Deya has done everything that her grandparents expect—attending Islamic school, never leaving the house without them, never associating with boys—but she resists Fareeda’s attempts to marry her off because she wants to go to college. Fareeda, however, shames her into silence by asking whether Deya really thinks they will allow her to “turn into an American” (Rum, 2019b, 189). Since shame, by definition, affects the “social self,” Deya’s fear of familial and social disapproval discourages her from speaking up for herself and against the culturally enforced silencing of women whose being and behaving does not conform to traditional Arab notions of femininity.

But everything changes when a woman drops off a business card for Deya in front of their house and the girl decides to skip school and meet her. What follows is the one scene in the novel in which a character does not experience shaming within the Arab American community, but outside it. Riding the subway alone for the first time in her life and wearing the hijab that is part of her school uniform, Deya observes the strangers around her as she is being observed herself:

They were Italian, Chinese, Korean, Mexican, Jamaican—every ethnicity Deya could possibly imagine—yet something about them seemed so American. What was it? Deya thought it was the way they spoke—their voices loud, or at least louder than hers. It was the way they stood confidently on the train, not apologizing for taking up the space. [. . .]

She could see the judgment brewing in their eyes. She could feel them observing how scared she was standing there, how unassuredly she moved, the garb she wore, and deciding instantly that they knew everything about her. Surely she was the victim of an oppressive culture. [. . .] The trouble was, regardless of what they saw, or how little they thought of her, in her own eyes Deya didn’t see herself much better. She was a soul torn down the middle, broken in two. Straddled and limited. Here or there, it didn’t matter. She didn’t belong. (Rum, 2019b, 107)

As Deya observes the diverse mix of ethnicities, she places all those commonly “hyphenated” Americans within the borders of American national belonging, while she experiences herself as fundamentally placeless. In so doing, she articulates the unique struggles of Muslim Americans whom Laila Lalami defines as “the ultimate Other of our age” (Lalami, 2011, 148). Her position as the ultimate Other is subsequently illustrated when the strangers on the subway, who belong to various ethnic minorities, coopt the othering gaze that is routinely reserved for “majority Americans.” As Deya is confronted with what Edward W. Said describes as the orientalist gaze, readers learn that fear and shame “clung in her throat” (Rum, 2019b, 108).^4^ The scene represents what Fetta calls “a scene of racialization, a stepped social practice [. . .] in which, with or without words, bodies impose social asymmetries through somatic expression” (Fetta, 2018, xv). It portrays how racial, gendered, and religious factors combine to produce anti‐Arab oppression within the broader U.S. American environment.^5^

Moreover, the passage also negotiates the internalization of the gaze of the other which causes Isra to have “a soul torn down the middle, broken in two” (Rum, 2019b, 107). This calls to mind W. E. B. Du Bois’s notion of double consciousness which suggests that African Americans are “born with a veil [. . .] measuring one’s soul by the tape of a world that looks on in amused contempt and pity” (Du Bois, 2014, 7). Deya’s hijab, a literal veil, attracts racial otherization in the public sphere, which in turn, adds to and intensifies feelings of gendered self‐worthlessness evoked within the walls of the character’s home.^6^ Of course, the tension inherent in double consciousness, and the act of measuring one’s soul through the judgment of the outside world, is also integral to the consciousness and pain of shame.

Deya’s unsettling trip on the subway finally leads her to Sarah, Fareeda’s rebellious daughter, who ran away from home because she was no longer a virgin and afraid of what would happen to her if her parents found out. While Sarah started a new life somewhere in the U.S., Fareeda made everyone believe that her daughter had married a man in Palestine, in order to save the family from the shame of having a “tainted” daughter. Knowing that running away means freedom at the cost of family and belonging, and in Isra’s case even life itself, Sarah does not encourage Deya to run away, but to stay with her sisters and speak up for all of them in front of their grandmother. By telling her own story and giving Deya the newspaper clipping about her mother’s murder, Sarah not only enables Deya to work through her memories of her parents, mourn, and potentially heal, she also encourages Deya to imagine and construct a path away from the compulsive repetitions of shaming and suffering that have affected the lives of women in their family. Before she meets Sarah, Deya “tucked her dreams away” because “she didn’t want to ruin her reputation in the community [. . .] Or worse, be disowned, banned from seeing her sisters, the only home and family she had ever known” (Rum, 2019b, 32). But with Sarah’s encouragement, Deya ultimately insists that she will not get married before she finishes college and Fareeda starts to listen. By taking a step towards a form of resistance that prioritizes the preservation of both the self and family over the preservation of a destructive culture of gendered shaming and silencing, the character of Deya imagines a strategy of individual and generational healing.

In the first part of the novel, readers are only given insight into Isra’s and Deya’s thoughts and feelings about their mother‐in‐law and grandmother, but as the narrative progresses Rum begins to weave in chapters that are focalized through Fareeda’s consciousness. Fareeda’s transition from a focalized character (who is the subject of the focalizing gaze of Isra and Deya) into a character focalizer (who is the focalizing subject herself) gives readers a more complex insight into her own inner life and the circumstances that have made her complicit in maintaining the cycle of gendered silence and shame. While the chapters focalized through Isra reveal her resentment of Fareeda, it also becomes clear that she admired her mother‐in‐law’s strength, thinking that the “world had made a warrior out of her” (Rum, 2019b, 184). Indeed, her life experiences—being relocated to a refugee camp when Israeli soldiers invaded Palestine, moving to a foreign land, “being pushed and pulled, from kitchen to kitchen, child to child” (p. 119)—have taught Fareeda her place in the world. Fareeda

was not surprised when her father came home and beat them mercilessly, the tragedy of the *Nakba* bulging in his veins. Nor was she surprised when he married her off to a man who beat her, too. How could he not, when they were so poor that their lives were filled with continuous shame? She knew that the suffering of women started in the suffering of men, that the bondages of one became the bondages of the other. Would the men in her life have battered her had they not been battered themselves? Fareeda doubted it, and it was this awareness of the hurt behind the hurt that had enabled her to see past Khaled’s [Fareeda’s husband] violence over the years and not let it destroy her. (p. 116)

Rum points to the Nakba—the ethnic cleansing and expulsion of hundreds of thousands of Palestinians from their homeland beginning in 1948—to provide a broader context for the intergenerational effects of traumatic experiences.^7^ Studies on the impact of refugee trauma, indeed, suggest that there is “an increased risk of adverse psychological outcomes and vulnerability to psychosocial stress within the next generation of refugee families,” which may lead to “greater risk of abuse and neglect” (Sangalang & Vang, 2017, 752). But while it is true that shame may lead to violence and anger, and while epigenetic research raises the possibility that traumatic effects may be intergenerationally inherited, Fareeda’s “explanation” that the batterers have been battered themselves cannot even begin to address the magnitude of the problem of domestic abuse in Arab American communities. Abuelezam, El‐Sayed, and Galea, for instance, suggest that “the structural causes of intimate partner violence in Arab American populations [. . .] include dependence on male relationships for stability and safety post immigration, patriarchal Arab culture, the lack of cultural support for seeking marital help outside of the family, and family honor, and blaming” (Abuelezam et al., 2018, 5). In an interview, Rum points out that she included passages like this in her novel because she did not want to portray Arab American men as “monster[s].” Instead, she intended to recognize “the further trauma of dislocation that impacts men who then take their frustrations out on the women in their lives” (Rum, 2019a). Rum’s aim to draw multidimensional male characters would, however, have profited from an insight into Khaled’s or Adam’s thoughts and emotions, rather than only offering overgeneralizing explanations for the men’s violence.

Even though Fareeda’s perception of the men in her life cannot replace the subjectivity of their own perspective, the inclusion of chapters that are focalized through her perspective does complicate readers’ interpretation of Fareeda herself. The insight into her own mind reveals that Fareeda has routinely denied confronting the traumas of her past by pushing unwelcome “thoughts away” (Rum, 2019b, 116). But when Deya confronts Fareeda with the truths—Isra’s death at the hands of Adam and the fate of her daughter Sarah—Fareeda tells her that she lied to save all of them from shame. Talking about this for the first time to someone else, she realizes that “She was so afraid of the shame the family would face” that she erased her daughter from her life (p. 268). Fareeda reveals how her own shame about giving birth to two twin girls—who tragically died from malnutrition in the refugee camp—made her dangerously complicit in patriarchal practices of female oppression and abuse. Rather than empathizing with the struggles of the women in her life, Fareeda kept shaming others because “shame could grow and morph and swallow someone until she had no choice but to pass it along so that she wasn’t forced to bear it alone” (p. 280). Yet, after acknowledging the life‐changing traumatic experience of her own life, Fareeda is “feeling a weight about to come undone” (p. 265). While her attempts to dissipate her own negative emotions have contributed to reinforcing the cross‐generational cycle of shame, the ending promotes the sharing of felt experience through storytelling as a powerful strategy of emotion regulation, resistance, and healing.

## 4 CONCLUSION

At the age of nineteen, Etaf Rum herself entered into an abusive arranged marriage. With writing came the realization that she could no longer stay silent about the realities of her own life. After completing her novel about gendered shaming and violence within the Arab American community—thus having committed “the ultimate shame” (Rum, 2019b, 1)—she left her husband and got a divorce. While *A Woman Is No Man* draws attention to her own story and the stories of so many other women whose voices are routinely silenced, resistance always comes with a price. The more personal consequences of addressing familial and inter‐community domestic abuse include the loss of a sense of family and community, whereas the more collective risk of addressing gender oppression within the Arab American community lies in confirming existing stereotypes.^8^

In the past decades, there has been a proliferation of Arab American literature which is as diverse as the Arab American community itself. Arab American authors who portray negative aspects of Arab American life have, however, often been labeled as traitors to the community, because they play into—and possibly even solidify—already widespread anti‐Arab and anti‐Muslim sentiment. For better or worse, Rum does not use literary space to “correct” mainstream (Western) conceptions about Arab Americans. Instead, she uses it to critique inter‐community problems of patriarchy, gender‐based violence, and shaming. Not only does she draw attention to the impact that the complex, self‐conscious emotion of shame may have for the lived experiences of many Arab American women, she also foregrounds the importance of breaking cycles of silence and shame—be they familial or literary. Ultimately, *A Woman Is No Man*, like any other “single story,” should not be taken to define and homogenize the very diverse experiences of Arab American women.

My reading of the novel uncovers the ways in which Rum’s literary representation of the experiences of three Arab American women explores the “shameful” underpinnings of Arab American patriarchy. The narrative revelation of the negative and self‐conscious emotion of shame through the characters’ individual consciousness and their social interactions discloses not only the relations between the elicitation, experience, expression, and regulation of shame. It also highlights the socio‐cultural and gendered power structures that influence emotion and behavior. Since narratives are “important indicators of what we feel and why—and perhaps if and how things should be changed” (Kim, 2013, 26), cognitive approaches to narrative emotion are especially well‐suited to analyze the affective structures that shape both literary representation and lived experience.

## ENDNOTES

^1^
See, for example, Aldama’s *Your Brain on Latino Comics* (2009), Kim’s *On Anger* (2013), Weik von Mossner’s *Cosmopolitan Minds* (2014), and Fetta’s *Shaming into Brown* (2018).

^2^
For psychological research on the correlation between shame‐proneness and alcohol and drug use see, for example, Dearing, Stuewig, and Tangney (2005).

^3^
Emerging research in epigenetics suggests that parental trauma effects may be intergenerationally transmitted through changes in gene expression, but not in the DNA sequence itself (e.g. Yehuda & Lehrner, 2018).

^4^
Said argues that “The West is the actor, the Orient the passive reactor. The West is the spectator, the judge and jury, of every facet of Oriental behavior” (Said, 2003, 109).

^5^
The novel’s portrayal of a strictly patriarchal and conservative but not “devout Muslim family,” whose strict norms and rules are not “based on religion at all only Arab propriety” (Rum, 2019b, 83), challenges common and dangerous forms of Western, anti‐Muslim rhetoric which equate Islam with women’s oppression.

^6^
The scene offers a representation of the hijab as “shutting Muslim girls and women out from a vast world” (Jarmakani, 2012, 162). The hijab is, however, also an important and empowering symbol of Muslim women’s identity. Jarmakani notes that “despite the tired dichotomies that seem to endlessly flutter around the hijab, it has functioned for some as a means of negotiating a ‘third space’ that works to dislodge the metaphorical binaries (i.e. reveal/cover, veil/unveil, submissive/threatening) that seek to discipline Muslim women and girls” (p. 162).

^7^
See, for example, Nur Masalha’s *The Palestine Nakba: Decolonising History, Narrating the Subaltern, Reclaiming Memory* (2012).

^8^
Whereas Abu‐Jaber points out that Rum’s novel is “a tale as rich and varied as America itself” which “complicates and deepens the Arab American story” (Abu‐Jaber, 2019), the literary representation of controversial inter‐community issues remains highly contested. In a 2002 interview, Abu‐Jaber responded to the criticism directed at her own writing in relation to the “tense fearfulness” that Arab Americans have “about the way that they and their culture are written about.” For her the choice is clear: “If you silence yourself, if you try to be good, if you try to be polite, or toe a party line, you end up paying for that in the long run. You pay for it […] with your homeland, or with your soul, or with your artistic vision” (Abu‐Jaber, 2002).
